# The hidden teeth of sloths: evolutionary vestiges and the development of a simplified dentition

**DOI:** 10.1038/srep27763

**Published:** 2016-06-14

**Authors:** Lionel Hautier, Helder Gomes Rodrigues, Guillaume Billet, Robert J. Asher

**Affiliations:** 1 Institut des Sciences de l’Evolution de Montpellier, Université Montpellier, CNRS, IRD, EPHE, Cc 064; place Eugène Bataillon, 34095 Montpellier Cedex 5, France; 2 Sorbonne Universités, CR2P, UMR CNRS 7207, Univ Paris 06, Muséum national d’Histoire naturelle, 8 rue Buffon, 75005 Paris, France; 3 Mécanismes adaptatifs et évolution (MECADEV), UMR 7179, CNRS, Funevol team, Muséum national d’Histoire naturelle, 55 rue Buffon, Bat. Anatomie Comparée, CP 55, 75005 Paris, France.; 4 Department of Zoology, University of Cambridge, Downing St., Cambridge CB2 3EJ, UK

## Abstract

Xenarthrans are unique among mammals in retaining simplified teeth that are rootless and homodont, which makes it difficult to determine dental homologies. We apply computerized tomography to prenatal developmental series of extant sloths, *Bradypus* and *Choloepus*, to further elucidate the patterns of morphological variation in their dentition. We also propose new criteria based on sequences of dental mineralization, and the presence of vestigial teeth, to distinguish between caniniforms and postcaniniforms. We report for the first time the presence of vestigial incisors in *Bradypus*. We also show the presence of a vestigial tooth in front of the lower caniniform in both extant sloth genera and the existence of two generations for the upper caniniform in *Choloepus*. The study of their sequence of mineralization indicates that the lower and upper caniniform teeth are not homologous in sloths, and suggests that upper caniniforms are not homologous between the two extant sloth genera. Our results show that assessing the developmental processes and functional constraints remains crucial to understand the dental variations observed in sloths, and more generally, tooth class homology issues in mammals. Applied to the tooth row of all extinct sloths, these developmental data illuminate a potentially ancestral dental formula for sloths.

Like other xenarthrans (sloths, armadillos, and anteaters), living and extinct sloths (Folivora) depart from the rest of mammals by the simplified nature of their dentition. Teeth present in most xenarthran adults lack enamel and are usually homodont, ever-growing, tubular and primarily composed of orthodentine and vasodentine^1^, which makes it difficult to identify homologies with the teeth and cusps of other mammals. Both extant sloth genera are functionally monophyodont, and their dentition is generally considered to constitute a single set of permanent teeth^2^,^3^,^4^,^5^,^6^. The sloth dentition contrasts with the complete lack of teeth in anteaters, and the supernumerary teeth of armadillos. It mainly differs from that of other xenarthrans in showing a morphological distinction between caniniforms and molariforms, a difference based on the general morphology, occlusion, and position of their teeth.

Recent morphological and molecular phylogenetic analyses^7^,^8^,^9^,^10^ suggested that the two modern genera are only distantly related, with a divergence time that could be as long as 30 million years ago^11^. Despite this independent evolutionary history, both two-toed and three-toed sloths display identical dental formulae with five upper and four lower teeth, as do the majority of extinct sloth genera^1^,^10^. This apparent stability in number associated with the differentiation of the tooth row observed in extant forms masks a complex evolution of the dentition in folivorans (*i.e*., modern sloths end extinct gravigrade sloths). *Bradypus* shows a closely fit toothrow, lacking diastema with each tooth showing a peg-like morphology. *Choloepus* displays an enlarged, chisel-shaped caniniform at the front of the dentition and isolated from the molariforms by a diastema^1^,^12^.

While the intriguing nature of the xenarthran teeth has attracted a lot of attention, few studies have focused on the development of the whole dentition^13^, especially in sloths. Early workers have only described isolated foetuses of sloths or focused on the developmental sequence of their skeleton^14^,^15^. However, these do not detail the development of the teeth or provide a comparative basis upon which to analyse possible homologies with the dentition of other mammals. Using a large dataset of scanned foetuses of sloths, we provide data on xenarthran prenatal dental ontogeny, identify some developmental criteria with which to recognize homologies with other mammalian teeth, and propose a new hypothesis for the development of heterodonty in sloths.

## Results

A terminology specific to sloths has been used to avoid confusion and in order to draw reliable comparisons (S1): pmx stands for premaxilla; d stands for deciduous teeth; cf and mf stand for lower caniniforms and molariforms respectively, while Cf and Mf stand for upper teeth; lower loci 1–3 and upper loci 1–4 involve functional molariform teeth; v and V stand for vestigial lower and upper teeth respectively (i.e., these loci are absent in adults).

### Prenatal dental development in three-toed sloths

**Figure 1:**
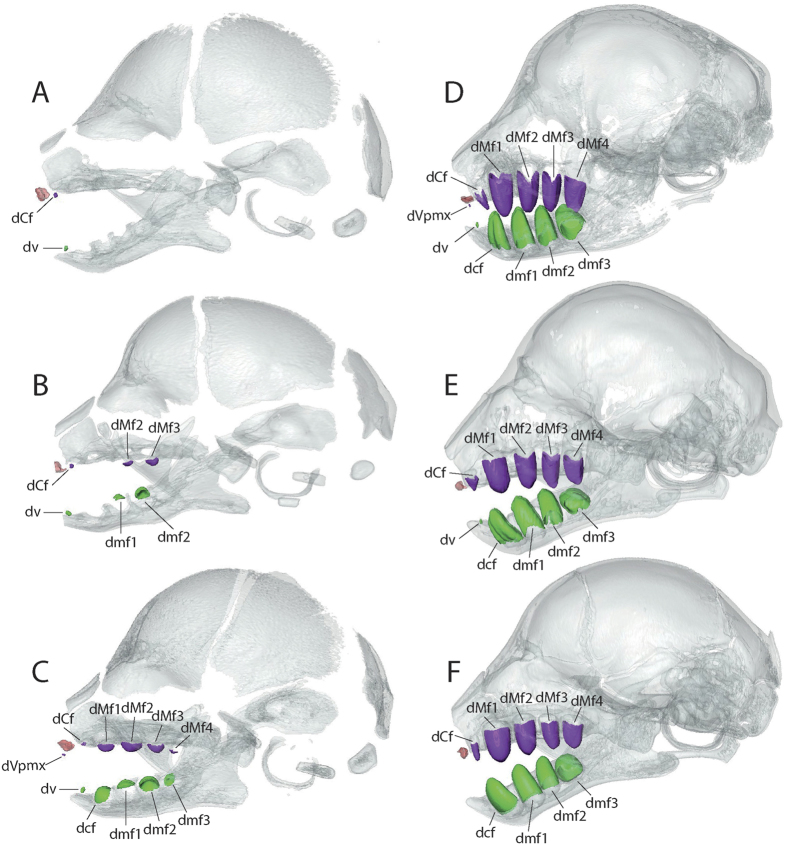
Lateral view of three-dimensional reconstruction of CT-scans of skull in the three-toed sloth *Bradypus*. (**A**) *Bradypus variegatus* (ZMB 33812), SL = 23 mm; (**B**) *Bradypus variegatus* (ZMB 41122), SL = 26 mm; (**C**) *Bradypus variegatus* (MNHN-ZM-MO-1995-326A), SL = 26 mm; (**D**) *Bradypus variegatus* (ZMB 41120), SL = 42 mm; (**E**) *Bradypus tridactylus* (BMNH 52-1173), SL = 42 mm; (**F**) *Bradypus sp.* (MNHN-ZM-MO-1995-327), SL = 38 mm. Upper teeth are in violet; lower teeth are in green; premaxillary bone is in red.

**Figure 2:**
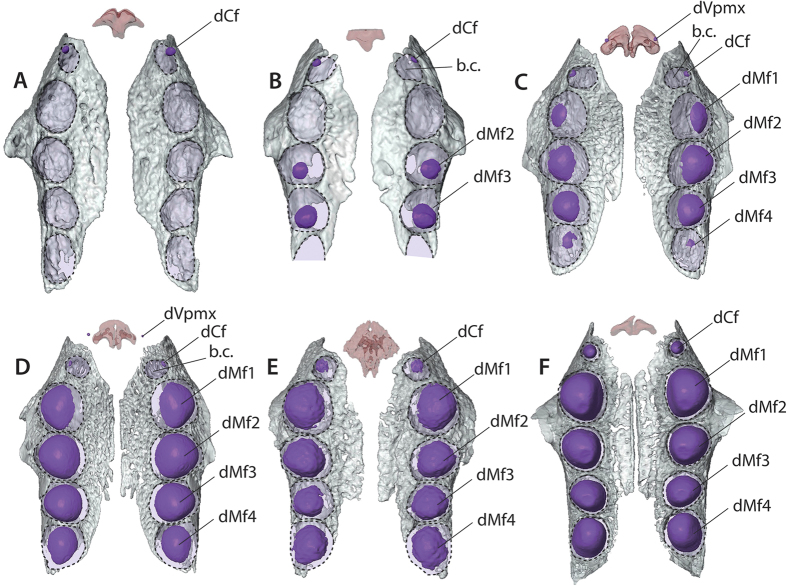
Palatal view of three-dimensional reconstruction of the maxillary bones in early developmental stages of the three-toed sloth *Bradypus*. (**A**) *Bradypus variegatus* (ZMB 33812), SL = 23 mm; (**B**) *Bradypus variegatus* (ZMB 41122), SL = 26 mm; (**C**) *Bradypus variegatus* (MNHN-ZM-MO-1995-326A), SL = 26 mm; (**D**) *Bradypus variegatus* (MNHN-ZM-MO-1995-326B), SL = 30 mm; (**E**) *Bradypus sp.* (MNHN-ZM-MO-1902-325), SL = 30 mm; (**F**) *Bradypus sp.* (MNHN-ZM-MO-1995-327), SL = 38 mm. Upper teeth are in violet; premaxillary bone is in red. Dashed lines represent dental alveoli. *Abbreviations*: b.c., bony crypt.

The sequence of prenatal dental eruption in *Bradypus* is well resolved with 18 specimens that represent a variety of developmental stages. Eleven of 18 foetuses display a number of developing teeth different from those observed in adults, which (as noted previously) are characterized by five upper and four lower teeth (*i.e.*, 5/4). All the alveoli of the adult teeth are present early during dental development, but lack teeth. This implies that dental buds are developing but not yet mineralizing; these buds cannot be directly observed because soft tissues are difficult to detect using X-ray microtomography without soft-tissue staining. In the youngest specimen (ZMB 33812, SL = 23.7 mm, Figs 1A and 2A), only the mesialmost pairs of teeth are mineralizing on the maxillary and the dentary (dCf and dv), meaning that dentine formation has started. In contrast to other teeth, the first pair of uppers (dCf) to appear are not centered in their alveolus, but sit off-centre in the anterolateral corner of the alveolus. The dCf is the first locus to mineralize, but its size does not change drastically during the first stages. Other teeth mineralize after dCf, but grow more quickly (Figs 1A–D and 2). The second youngest specimen (ZMB 41122, SL = 25.9 mm, Figs 1B and 2B) shows mineralized dCf, dMf2, dMf3, dv, dmf1, and dmf2, with empty alveoli (*i.e.*, teeth not yet mineralizing) at the dMf1, dMf4, dcf, and dmf3 loci. All the alveoli include mineralized teeth in subsequent stages.

More importantly, four of 18 specimens have six upper and five lower teeth; they show an extra pair of teeth on the premaxilla (dVpmx, Fig. 1C,D), which correspond to rudimentary incisors, absent in the adults. These incisors can be retained until relatively late in development (*e.g.*, ZMB 41120, SL = 41.84 mm, Fig. 1D), but are resorbed before birth. Five specimens have a dental formula composed of five upper and five lower teeth. The first pair of lower teeth (dv), just mesial to the lower caniniform (dcf), is also resorbed during development, likely after the small incisors, and is absent in later stages. Both extra upper and lower teeth (dVpmx and dv) are apparent on both right and left sides, they do not have visible extension of the root, and are never associated with alveoli. We observed no major dental differences between *B. variegatus* and *B. tridactylus*, both of which exhibited similar morphology at comparable stages.

### Prenatal dental development in two-toed sloths

**Figure 3:**
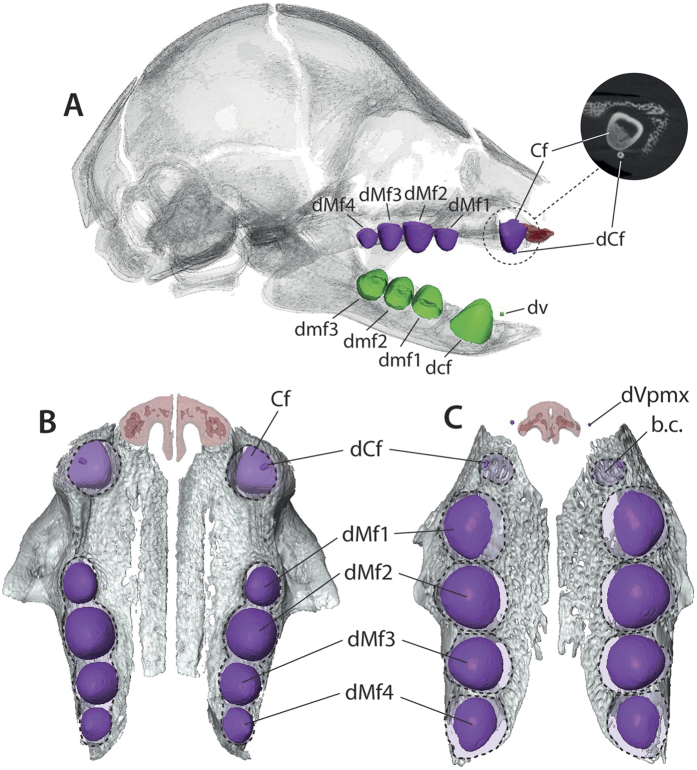
A comparison of the tooth rows in *Choloepus* and *Bradypus*. (**A**) lateral view of the skull of *Choloepus*. (**B,C**) palatal views of the tooth rows in *C. didactylus* ((**B**) MNHN-ZM-MO-1882-625, SL = 45 mm) and *B. variegatus* ((**C**) MNHN-ZM-MO-1995-326B, SL = 30 mm). Note the similar (off-centre) position of the dCf in the mesialmost alveolus.

The skull length (see S2 for measurements) and number of discrete ossification centres of the cranium indicate that most of the specimens of *Choloepus* correspond to relatively late stages compared to *Bradypus*. However, as for *Bradypus*, the number of teeth varies greatly among our specimens and differs from the morphology observed in adult *Choloepus*. While the adult dental formula is 5/4, the foetuses showed either five upper and five lower teeth (60% of the cases) or six upper and five lower teeth (40% of the cases). All specimens display an extra tooth on the mandible in front of the functional adult tooth row (dv), i.e., in front of the lower caniniforms (dcf). Two younger specimens also show an extra tooth in the maxilla (dCf; Fig. 3A) in front of the upper caniniforms (Cf); these extra teeth are located in the same alveoli as Cf and are oriented mesio-buccally (Fig. 3A,B). As observed for *Bradypus*, all extra teeth are present on both right and left sides; however, in contrast to *Bradypus*, no vestigial incisor (dVpmx) was observed in the premaxilla of *Choloepus*, although we cannot rule out its presence in specimens younger than those in our sample. No extra teeth were detected at the level of the diastema that separates the mesialmost functional tooth from the molariforms in any of the specimens studied. As observed in *Bradypus*, the lower teeth (dv) do not have visible extension of the root and are never associated with alveoli, in contrast to the teeth present in adults. We observed no major dental differences between the two species of *Choloepus*.

## Discussion

### A history of prenatal dental development in extant sloths

For Böker^16^, “*sloths will always remain the poor sibling of comparative anatomy, because it is neither possible to obtain complete anatomical series, nor does the possibility currently exist to gain good insights from paleontology. Only ontogeny promises good prospects to enlighten the history of sloths’ modifications. Yet this does not promise an easy path either, because not only are important developmental stages very difficult to find, but even when embryos are available, they show that development of key anatomical structures seems to occur at very early stages*” (in German in the text). Comparisons of tooth development in sloths suggests that, for at least some anatomical regions, Böker is correct that ontogeny is a key source of information about homology. However, he is too pessimistic that sloths will remain poorly understood compared to other animals. Extant and extinct sloths display quite homodont teeth, which makes it difficult to determine dental homologies. Prenatal dental development in sloths shows teeth that are cone-shaped and monocuspid (see also^17^,^18^, and demonstrates that robust hypotheses of homologies cannot be drawn based on occlusal patterns alone (*e.g.*^19^,^20^), which simply result from rapid wear (*i.e.*, cusp-like pattern). However, our data show that *Bradypus* and *Choloepus* display several pairs of supernumary teeth in the mesial part of their dentition during prenatal ontogeny. This is consistent with previous, anecdotal accounts of dental “anomalies” in sloths^2^,^6^,^17^,^21^,^22^,^23^. Brandts^21^ (cited in Röse^22^) reported the first record of vestigial lower teeth in *Bradypus* and believed them to be canines. Parker^2^ observed similar vestiges in *Choloepus* embryos, which made him recognize the lower caniniform as a premolar locus. Gervais^17^ seemed unaware of Brandts’ reference when he described the presence of vestigial teeth in the mandible of a foetus of *Bradypus*, which he considered to be incisors based on their closeness to the symphysis as well as their position compared to the caniniforms. Simon^23^ reached similar conclusions with two foetuses of *Bradypus* (CRL = 23.5 and 24.2 cm); he also proposed, probably for the first time, the non-homology between the lower and upper caniniforms based on dental eruption sequences (the upper caniniform erupting much later than the upper molariform and the lower caniniform).

### Occurrence of vestigial teeth and dental anomalies in sloths

Our data show that *Bradypus* and *Choloepus* display several pairs of supernumary teeth in the mesial part of their dentition during prenatal ontogeny. This is consistent with previous, anecdotal accounts of dental “anomalies” in sloths. High-resolution X-ray computed tomography on a large number of foetuses demonstrated that these extra teeth cannot simply be explained by individual variation that occurs exceptionally within a population. Such methods also revealed that these teeth lack typical roots and do not develop in clearly individualized alveoli, unlike other teeth. More importantly, these vestigial teeth occur on both right and left sides, which is rarely the case with sloth dental anomalies^12^. Sloths display fewer dental anomalies compared to other mammals (*i.e.*, 2.4% of adult specimens observed by McAfee^12^ exhibited any sort of anomalies), and increases in tooth number (*i.e.*, hyperdontia) occur at a much lower rate than reductions^12^. *Bradypus* is more prone to lose teeth and all cases of tooth loss involve the dCf^12^, which could be logically expected since it is the most reduced tooth of the dentition^24^. Interestingly, nearly all of the anomalies observed in adults (unpaired hyperdontia and anodontia) affected the upper dentition in sloths^12^ while vestigial teeth were most consistently observed on the mandible. In fact, the only paired hyperdontia anomaly ever reported on the mandible (Fig. 1C)^12^ probably corresponds to a specimen that failed to resorb the mesial vestigial teeth (dcf) observed in the foetuses. This reinforces the idea that the mineralization and resorption of the vestigial teeth is an integral part of prenatal dental development in sloths. All of these teeth (dVpmx in *Bradypus*, dCf in *Choloepus*, and dv in both) correspond to the definition of vestigial structures given by Peterkova *et al*.^25^; they occur transiently during development in all members of a population, and on occasion persist into maturity. While both extant genera have similar development of the lower teeth, including the mineralization of paired mesial vestigial teeth (dv), differences are evident on the upper jaw, with *Bradypus* displaying premaxillary vestigial teeth and *Choloepus* maxillary but no premaxillary vestigial teeth.

### First evidence of tooth replacement in sloths

Vestigial teeth were observed in the maxillae of *Choloepus* (dCf). These vestigial teeth are very close to the caniniform teeth (Cf). They are located in the same alveolus, and appear apically with respect to the Cf (Fig. 3A,B), as expected in vertical dental replacement (*e.g.*^26^,^27^). Determining the epithelial connections of teeth during early developmental stages provides the best criterion for defining the deciduous or permanent homologies of individual teeth^28^,^29^, but unstained CT data do not convey this information on the differentiation of the dental lamina. However, based on positional data we interpret the vestigial upper tooth (dCf) and the caniniform (Cf) in *Choloepus* as deciduous and permanent teeth of the same locus^30^. Such an occurrence of non-functional vestigial deciduous teeth, rapidly replaced by permanent teeth, has already been reported in other mammalian groups such as marsupials (e.g. *Perameles*^29^), soricids (*e.g.*, *Sorex*, *Suncus*^27^,^31^), and mustelids (*e.g.*, *Mephitis*, *Enhydra*^32^,^33^). The presence of two dental generations in a folivoran is here reported for the first time. Our results hint at diphyodonty for at least one locus in sloths (*i.e.*, the caniniform in *Choloepus*), and are consistent with the expectation that ancestral xenarthrans possessed tooth replacement as typical for mammals. It can then be stated that the diphyodonty is a symplesiomorphy of the Xenarthra, as it is shared by the extant sloth *Choloepus* and the armadillo *Dasypus*^13^,^34^.

### Dental homologies between sloths

When considering the dental mineralization sequence in *Choloepus*, homologies are not obvious between the upper and lower tooth rows. This is partly due to the limited resolution of our ontogenetic sequence and the lack of data on early developmental stages for this genus. The youngest specimen already shows advanced stage of mineralization for the whole dentition. This might explain why vestigial incisors (dVpmx), rapidly resorbed in *Bradypus*, are not observed in *Choloepus*.

In contrast, the different ontogenetic stages of *Bradypus* enable precise hypotheses of dental homologies in extant sloths. Upper caniniforms (dCf) and lower vestigial (dv) teeth are the first teeth to start their mineralization in *Bradypus* (Fig. 1A–C) and probably belong to the same locus since they do so simultaneously^35^. The similar development of the dCf in *Choloepus* and in *Bradypus* (Fig. 3B,C), both in terms of size and position in the alveolus, and the similar early stages of development between dCf and dv in *Bradypus*, allow us to hypothesise that upper and lower vestigial mineralized buds of *Choloepus* (dCf and dv) are homologous. Such an explanation would imply that the upper caniniforms are not homologous in the two extant genera of sloths, with adults *Choloepus* showing a permanent caniniform (Cf) for that locus while adults *Bradypus* retain a deciduous caniniform (dCf). Following this hypothesis, the deciduous upper teeth present at a vestigial state in *Choloepus* would be functional in *Bradypus* in concert with an absence of a permanent generation for that locus. The large bony crypt long observed during the mineralization of the dCf of *Bradypus* would then represent an embryological holdover when the permanent tooth primordia (Cf) was still activated for that locus. Such an assumption is supported by a case of bilateral anomaly in *Bradypus*^12^ (Fig. 3A) involving both occurrences of mesial dCf and a distally large Cf, which corresponds to the configuration observed in the foetal series of *Choloepus*.

Alternatively, rather than a retained deciduous caniniform in adult *Bradypus*, it could be proposed that succession at this locus is not represented in our ontogenetic series for that genus. Following this alternative hypothesis, the upper functional caniniforms of both genera are homologous and correspond to permanent teeth. Then, dCf observed during the ontogeny of *Bradypus* would correspond to a vestigial deciduous canine that would eventually be replaced later on by a permanent tooth (Cf). This would imply that we are missing several early developmental events in *Bradypus*, between putative mineralization of Cf and reabsorption of dCf, which appears unlikely considering that we were able to trace the evolution in shape and size of the outline of the mesialmost alveolus (Fig. 2) and that *Bradypus* is the best-sampled genus in terms of the number of differently sized stages. The first hypothesis is thus preferred here.

### Simplified dentition *vs* the mammalian tooth row

Upper caniniforms (dCf) and lower vestigial (dv) teeth are the first teeth to start their mineralization in *Bradypus* (Figs 1A and 2A). Following the hypothesis that dCf and dv represent homologous deciduous teeth in *Choloepus* and *Bradypus*, these teeth are deciduous canines since the dC is one of the earliest tooth germs to differentiate in eutherian mammals^29^,^35^,^36^. The extreme mesial position of dCf on the maxillary bone (Figs 1 and 2), near the suture with the premaxilla, also provides further support in favour of this attribution.

The mineralization of dCf and dv is followed by the second and third upper molariforms (dMf2-3), which mineralize simultaneously with the first and second lower molariforms (dmf1-2). Such development is reminiscent of the initiation of distal milk premolars in mammals (*e.g.*, dP3-4^28^,^29^) although the proposed sequence is only based on two foetal *Bradypus* specimens. The vestigial premaxillary teeth (dVpmx) of *Bradypus* likely correspond to vestigial incisors that are resorbed during development and are never observed in adult specimens, not even as anomalies^12^. The relative timing of mineralization of the dVpmx, first upper molariform (dMf1), and lower caniniform (dcf) cannot be precisely determined based on our dataset. However, their mineralization likely occurs shortly before that of the distalmost upper and lower molariform teeth (dMf4 and dmf3) since the latter only show an incipient mineralization in the youngest specimen with evidence bearing on that locus (specimen MNHN CG 1995-326A, Figs 1C and 2C). If the general trends observed when studying the mammalian dental mineralization sequence (*e.g.*^28^,^29^,^37^) are valid for sloths, the last molariforms (dMf4 and dmf3) should be considered as first molars, preceded by three deciduous premolars (dMf1-3; dcf-dmf2) and a deciduous canine (dCf; dv). Notably, the early diverging living cingulate (*Dasypus*) has also been interpreted as exhibiting a single, unreplaced M1 locus in each jaw quadrant, preceded by replaced premolariforms and possibly a canine locus^34^. However, in contrast to *Dasypus*, no known folivoran shows replacement of functional milk teeth, and there is no empirical evidence to support this hypothesis since the possibility of a violation of the “normal” mammalian sequence cannot be entirely ruled out. The sloth dental formula might include supernumary teeth, as present for instance among the premolars of cingulates^13^,^34^ or in Mesozoic groups like docodonts or morganucodonts^38^.

In any case, these results support the assumption that the upper caniniforms present in adult *Bradypus* likely represent canines and that the upper and lower caniniforms (dCf, dcf) are not homologous since they mineralize at very different times during ontogeny^35^. The dcf can then be considered as a premolar locus, which might be homologous to dMf1 when compared their timing of mineralization. Such a hypothesis of non-homology between dCf and dcf was proposed early on and stemmed mainly from the fact that the upper caniniforms in sloths occlude with the mesial surface of the lower caniniforms, while upper canines occlude with the distal edge of the lower canines in other placentals^3^,^24^. Simon^23^ also noted that upper caniniforms of *Bradypus* erupt well after the lower caniniforms, although some studies on both extinct and extant mammals prefer developmental prenatal dental data over eruption sequences to establish dental homologies^29^,^35^.

### Reconstructing the ancestral dental formula of sloths

Most fossil sloths show a very similar number of teeth compared to adult specimens of extant species with five upper and four lower teeth. Except for dental anomalies^12^ and for the dubious fossil sloth *Entelops*^39^,^40^, this number is never exceeded in extinct folivorans (S3). Some mylodontids and nothrotheriids show a reduction in tooth number with a loss of upper caniniforms^10^ (S3). It is therefore parsimonious to propose an ancestral dental formula of five upper and four lower teeth for Folivora^10^ and match our reconstruction of the sloth ancestral dental formula (S3). Our developmental data offer new evidence for the loss of teeth during the evolutionary history of sloths in showing that some loci have been retained in foetal stages of extant forms. The vestigial upper incisors found in *Bradypus* embryos was never reported in any other sloth, but may have been present in the earliest folivorans. The lower vestigial tooth dv was reported in both *Bradypus* and *Choloepus*. Given the phylogenetic distance between the two genera^7^,^8^,^9^,^10^, it is likely that such a vestige was also present in early ontogenetic stages of the most recent common ancestor of Folivora. This idea is corroborated by the rare occurrence of teeth at a similar position in some fossil sloths^41^,^42^,^43^.

We showed that the functional upper caniniforms are very different in the two genera: *Choloepus* shows an ephemeral and tiny mineralized bud of dCf associated with a massive caniniform Cf, whereas *Bradypus* shows a moderately-sized, peg-like tooth dCf. Intermediate stages between these extreme patterns may well have occurred in fossil sloths and would have shown the succession of a Cf to a well-mineralized dCf. However, evidence for such a succession (for instance an erupting Cf in juvenile or subadult stages) remains unknown in the fossil record of sloths. This absence might lie in the scarcity of well-documented ontogenetic series for fossil sloths (*e.g.*^18^), although relatively few subadult stages are needed to document a succession of dental generations (*e.g.*^44^). So far, no succession of dental generations at the Cf locus has been observed in megatherioids, one of the potential allies to *Bradypus*^9^,^45^. Another explanation for the absence of dental replacement at the Cf locus in the fossil record of sloths may actually lie in the potential autapomorphic condition of the pattern observed in *Bradypus*. This genus is thought to represent a paedomorphic lineage when compared its skull morphology to other folivorans^46^,^47^. The retention of the dCf and absence of functional Cf in adults supports the concept of a paedomorphic *Bradypus* and could constitute another retained juvenile feature. If the retention and subsequent growth of dCf are unique to *Bradypus*, it is not surprising that no intermediate stage was found in the fossil record as close fossil relatives of three-toed sloths remain virtually unknown^45^. A highly autapomorphic condition in *Bradypus* can also account for many morphological discrepancies and could have erased or modified several inherited folivoran synapomorphies in this genus; this could explain why it is retrieved as fully basal^10^ while it might instead be more apically nested within the folivoran clade^9^,^12^.

In *Bradypus*, the retention of a short rostrum associated with the development of a large dMf1 may have inhibited the development and mineralization of a large permanent caniniform (Cf, Fig. 2D). This ontogenetic pattern gives room for a complete mineralization of the deciduous caniniform (dCf), which remains reduced (Fig. 2E). The growth of dCf seems to be “reactivated” only when the mineralization of all other teeth is well underway (Fig. 2E); only then does it quickly acquire its adult size. Interestingly, such a development of vestigial teeth that recover functionality has been proposed in a few mammalian species that show a reduced and simplified dentition, explained by minor developmental modifications (*e.g.*, frequent recovering of dP4 in the murine rodent, *Rhynchomys*^48^). This lends further credence to the findings of Simon^23^ who noticed the late eruption of the upper caniniforms compared to the lower caniniforms and all molariforms in *Bradypus*.

Following the hypothesis of an autapomorphic condition in *Bradypus*, the small dCf mineralized bud observed during the ontogeny of both extant genera might represent an ancestral feature for Folivora. Most of the diversity in shape of the “caniniforms” observed during the evolutionary history of sloths (*i.e.*, caniniform, incisiform, peg-like, entirely absent; see S3) could then originate from a permanent Cf, as in *Choloepus*. A large caniniform is present in earliest fossil sloths like *Octodontotherium*^49^ or *Pseudoglyptodon*^50^, and contrasts with our reconstruction of the sloth ancestral dental formula (S3) that is ultimately influenced by the basal rooting position of *Bradypus* on sloth phylogeny. Our results illuminate a potentially different ancestral dental formula for sloths that challenges the traditional assumption that the bradypodid tooth row is primitive and megalonychid dental features derived^10^. Such a hypothesis also mitigates the potential weight of dental features in phylogenetical and systematic studies, especially those related to the size and shape of the caniniforms. As a matter of fact, Gaudin^10^ (p. 275) commented that “*the family* [Megalonychidae] *is united largely by features associated with the caniniform first upper and lower teeth*”.

In sloths, the diversification in shape of the mesialmost teeth is frequently associated with a variation in rostral length and the presence of a pre and/or post diastema (S3). Such a diastema, which is often considered as a toothless gap, could challenge the homology of the teeth between taxa. However, the intercalation of additional teeth in the diastema, as observed in armadillos^13^, seems unlikely because of the relative stability of the dental formula in the sloth fossil record (S3). Our observations are consistent with McAfee’s view on the development of the diastema in *Choloepus*^12^, which he proposed could result from an increase of skull length and migration of mesial teeth rather than a loss of teeth between the caniniforms (dCf/Cf-dcf) and molariforms (dMf1-dmf1). The lesser development of the diastema in the youngest stages of *Choloepus* and the complete absence of vestigial teeth at its level are also in line with this assertion.

In conclusion, we showed that vestigial teeth are informative in understanding dental homologies, especially in assessing the deciduous or successional nature of individual teeth. Our developmental data for extant sloths bear directly on the claim that their lower caniniform teeth are not homologous to canines of other mammals and that upper caniniforms are not homologous between the two-toed and the three-toed sloths. These results underline that defining dental homologies in extant and extinct sloths is complex and that, where possible, characters based on dental features should be augmented with developmental data to ensure proper homology assessment. Development of discrete shapes and functional domains in the tooth row is governed by developmental processes that are still poorly known in mammals and for which further investigations on non-model mammals, such as sloths, are timely and topical.

## Methods

We sampled material from collections of the Museum für Naturkunde Berlin (ZMB), the Natural History Museum of London (BMNH), the Muséum National d’Histoire Naturelle in Paris (MNHN), and the Institut Royal des Sciences Naturelles de Belgique in Brussels (IRSNB). A total of 25 unsexed sloth foetuses were examined, representing four species of both extant genera: *Bradypus tridactylus*, *Bradypus variegatus*, *Choloepus didactylus*, and *Choloepus hoffmanni*^51^. Species identification was based on collection data (especially geographical origin) and cranial anatomy^51^,^52^ and was possible for 17 of our 25 specimens (S2). They range in size from 70 to 200 mm crown rump length (CRL), measured from the vertex of the skull to the base of the tail. Collections of such non-model organisms often include specimens collected decades ago and invariably lack data on individual age. Assignment to a relative developmental stage was based on the Skull Length (SL) and the number of discrete ossification centres throughout the skeleton^15^,^53^,^54^.

### 3-D data acquisition

Skulls were imaged using high-resolution microtomography (μCT) at the Helmholtz Zentrum (Berlin, Germany), at the Natural History Museum (London, UK), at the AST-RX platform MNHN (Paris, France), and at VISCOM SARL (Saint Ouen l’Aumône, France). This method allows 3D renderings of ossified tissues, as well as non-invasive virtual extractions of dental elements. Due to the scan resolution, we could not test for the putative presence of a small enamel cap at the tips of the forming teeth. These reconstruction and visualization were performed using stacks of digital CT images with the AVIZO 7.1 (Visualization Sciences Group) software. 3D reconstruction of the specimens were deposited in MorphoMuseum (http://www.morphomuseum.com/; M3#109 to M3#115) and Morph-D-base (https://www.morphdbase.de/).

### Reconstruction of the ancestral dental morphotype (S3)

The datamatrix of Gaudin^10^ was downloaded from Morphobank and the following characters of interest were selected for study: character n°2: dental formula; n°6: diastema; n°13: size of Cf ; n°14: size of cf; n°19: morphology of Cf/cf; n°21: position of Cf relative to the anterior edge of the maxilla. The cladogram corresponds to the topology of the strict consensus obtained by Gaudin^10^: Fig.1 when all characters were weighted equally. For our analysis of character optimizations, this cladogram was pruned in order to contain only sloth taxa (Folivora) (i.e., all non-folivoran successive outgroups originally included in Gaudin’s analysis^10^ were excluded). Parsimonious reconstruction of the hypothetical ancestral morphotype (S3) for the selected characters was undertaken on this reduced cladogram using the software Mesquite 2.75^55^.

## Additional Information

**How to cite this article**: Hautier, L. *et al*. The hidden teeth of sloths: evolutionary vestiges and the development of a simplified dentition. *Sci. Rep.* **6**, 27763; doi: 10.1038/srep27763 (2016).

## Supplementary Material

Supplementary Information

Supplementary Information

Supplementary Information
