# Supplementary material for: The hidden teeth of sloths: evolutionary vestiges and the development of a simplified dentition
Source: Sci Rep. 2016 Jun 14;6:27763. doi: 10.1038/srep27763 (PMC4906291; doi:10.1038/srep27763)
Supplement: Supplementary Information [file srep27763-s1.pdf]

Title: The hidden teeth of sloths: evolutionary vestiges and the development of a simplified dentition.

Authors

Lionel Hautier[1]

Helder Gomes Rodrigues[2,3]

Guillaume Billet[2]

Robert J. Asher[4]

Author affiliations

[1]Institut des Sciences de l'Evolution de Montpellier, Université Montpellier, CNRS, IRD, EPHE, Cc 064; place Eugène Bataillon, 34095 Montpellier Cedex 5, France.

[2] Sorbonne Universités, CR2P, UMR CNRS 7207, Univ Paris 06, Muséum national d'Histoire naturelle, 8 rue Buffon, 75005 Paris, France.

[3]Mécanismes adaptatifs et évolution (MECADEV), UMR 7179, CNRS, Funevol team, Muséum national d'Histoire naturelle, 55 rue Buffon, Bat. Anatomie Comparée, CP 55, 75005 Paris, France.

[4]Department of Zoology, University of Cambridge, Downing St., Cambridge CB2 3EJ, UK.

Corresponding authors

Lionel Hautier: lionel.hautier@univ-montp2.fr

S1 – Dental terminology used for the comparisons between *Choloepus* (A and B) and *Bradypus* (C and D). Upper teeth are in violet; lower teeth are in green; premaxillary bone is in red.

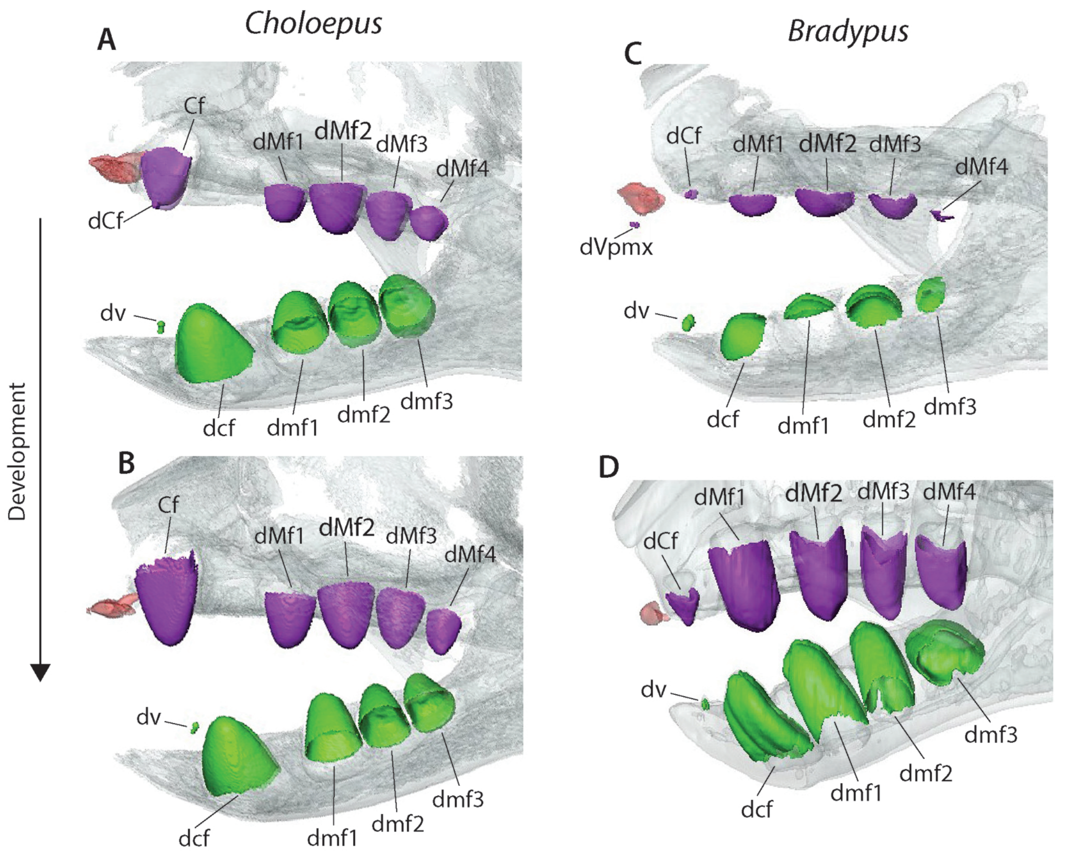
Choloepus
Bradypus
A
B
C
D
Development
Cf
dCf
dMf1
dMf2
dMf3
dMf4
dv
dcf
dmf1
dmf2
dmf3
Cf
dMf1
dMf2
dMf3
dMf4
dv
dcf
dmf1
dmf2
dmf3
dCf
dMf1
dMf2
dMf3
dMf4
dVpmx
dv
dcf
dmf1
dmf2
dmf3
dCf
dMf1
dMf2
dMf3
dMf4
dv
dcf
dmf1
dmf2
dmf3
