# Supplementary material for: The hidden teeth of sloths: evolutionary vestiges and the development of a simplified dentition
Source: Sci Rep. 2016 Jun 14;6:27763. doi: 10.1038/srep27763 (PMC4906291; doi:10.1038/srep27763)
Supplement: Supplementary Information [file srep27763-s2.pdf]

Title: The hidden teeth of sloths: evolutionary vestiges and the development of a simplified dentition.

Authors

Lionel Hautier[1]

Helder Gomes Rodrigues[2,3]

Guillaume Billet[2]

Robert J. Asher[4]

Author affiliations

[1]Institut des Sciences de l'Evolution de Montpellier, Université Montpellier, CNRS, IRD, EPHE, Cc 064; place Eugène Bataillon, 34095 Montpellier Cedex 5, France.

[2] Sorbonne Universités, CR2P, UMR CNRS 7207, Univ Paris 06, Muséum national d'Histoire naturelle, 8 rue Buffon, 75005 Paris, France.

[3]Mécanismes adaptatifs et évolution (MECADEV), UMR 7179, CNRS, Funevol team, Muséum national d'Histoire naturelle, 55 rue Buffon, Bat. Anatomie Comparée, CP 55, 75005 Paris, France.

[4]Department of Zoology, University of Cambridge, Downing St., Cambridge CB2 3EJ, UK.

Corresponding authors

Lionel Hautier: lionel.hautier@univ-montp2.fr

S2 – List of specimens, including the number of upper and lower teeth observed (functional and vestigial), the skull length taken on the 3D reconstruction, and the voxel size.

Specimen number	Genus	Species	Upper teeth	Lower teeth	Skull length	Voxel size (mm)
BMNH 52-1173	*Bradypus*	*tridactylus*	5	5	41.83	0.0766
BMNH 79-6-3	*Bradypus*	*tridactylus*	5	5	32.3	0.0528
BMNH1989-226	*Bradypus*	*tridactylus*	5	4	58.07	0.0991
BMNH 1994-6B	*Bradypus*	*tridactylus*	6	5	31.52	0.0435
BMNH 2010-89	*Bradypus*	*sp.*	5	4	41.71	0.0573
ZMB 91-95	*Bradypus*	*tridactylus*	5	5	40.91	0.0414
ZMB18834	*Bradypus*	*tridactylus*	5	4	34.94	0.0420
ZMB18835	*Bradypus*	*sp.*	5	4	37.81	0.0400
ZMB 33812	*Bradypus*	*variegatus*	1	1	23.73	0.0292
ZMB 41120	*Bradypus*	*variegatus*	6	5	41.84	0.0433
ZMB 41122	*Bradypus*	*variegatus*	3	3	25.86	0.0371
MNHN 1881-111	*Bradypus*	*tridactylus*	4	5	29.63	0.1080
MNHN1902-325	*Bradypus*	*sp.*	5	5	30.24	0.0700
MNHN1933-489	*Bradypus*	*sp.*	5	4	43.21	0.1400
MNHN 1995-326 A	*Bradypus*	*variegatus*	6	5	25.85	0.0283
MNHN 1995-326 B	*Bradypus*	*variegatus*	6	5	30.44	0.0356
MNHN 1995-327	*Bradypus*	*tridactylus?*	5	4	38.1	0.0571
MNHN 1918-18	*Bradypus*	*sp.*	5	4	49.71	0.1500
BMNH 72-10-24-1	*Choloepus*	*didactylus*	6	5	41.34	0.0268
BMNH 1863-12-19-2	*Choloepus*	*didactylus*	5	5	52.62	0.0991
BMNH 1882-625	*Choloepus*	*didactylus*	6	5	45.14	0.0250
ZM AC 1901-393	*Choloepus*	*didactylus*	5	5	44.44	0.0262
ZMB 49 49	*Choloepus*	*hoffmani*	5	5	49.23	0.0422
