# Supplementary material for: The hidden teeth of sloths: evolutionary vestiges and the development of a simplified dentition
Source: Sci Rep. 2016 Jun 14;6:27763. doi: 10.1038/srep27763 (PMC4906291; doi:10.1038/srep27763)
Supplement: Supplementary Information [file srep27763-s3.pdf]

Title: The hidden teeth of sloths: evolutionary vestiges and the development of a simplified dentition.

Authors

Lionel Hautier[1]

Helder Gomes Rodrigues[2,3]

Guillaume Billet[2]

Robert J. Asher[4]

Author affiliations

[1]Institut des Sciences de l'Evolution de Montpellier, Université Montpellier, CNRS, IRD, EPHE, Cc 064; place Eugène Bataillon, 34095 Montpellier Cedex 5, France.

[2] Sorbonne Universités, CR2P, UMR CNRS 7207, Univ Paris 06, Muséum national d'Histoire naturelle, 8 rue Buffon, 75005 Paris, France.

[3]Mécanismes adaptatifs et évolution (MECADEV), UMR 7179, CNRS, Funevol team, Muséum national d'Histoire naturelle, 55 rue Buffon, Bat. Anatomie Comparée, CP 55, 75005 Paris, France.

[4]Department of Zoology, University of Cambridge, Downing St., Cambridge CB2 3EJ, UK.

Corresponding authors

Lionel Hautier: lionel.hautier@univ-montp2.fr

S3 – **A**, a schematic representation of the tooth row in *Choloepus* and *Bradypus*, and a parsimonious reconstruction of the sloth ancestral dental morphotype. Dashed lines and question marks indicate ambiguous reconstruction for a given character (*i.e.*, state varies depending on the optimization mode[1]), as it is the case for the shape of the upper and lower caniniforms and the presence of an prediastema. **B**, Phylogeny of extinct and extant sloth genera [2] with their dental formulae. Only the differences in size and shape of the caniniform teeth were reported here. The dental characters presented here mainly follow the matrix of Gaudin[2] (*i.e.*, characters 2, 6, 13, 14, 19, and 21). Upper teeth are in violet; lower teeth are in green; caniniform teeth are in red; pre and post diastemas are represented by arrows. The colors of the clades (families) are as follows: red, Megalonychidae; yellow, Mylodontidae; blue, Megatheriidae; green, Nothrotheriidae. The names Cf/cf and Mf/mf only specify the loci, not the dental generation (e.g., Cf or dCf). Dashed lines represent ambiguous characters.

References

1. Swofford, D. L. & Maddison, W. P. Reconstructing ancestral character states under Wagner parsimony. *Math. Biosci.* **87,** 199–229 (1987).
2. Gaudin, T. J. Phylogenetic relationships among sloths (Mammalia , Xenarthra , Tardigrada): the craniodental evidence. *Zool. J. Linn. Soc.* **140,** 255–305 (2004).

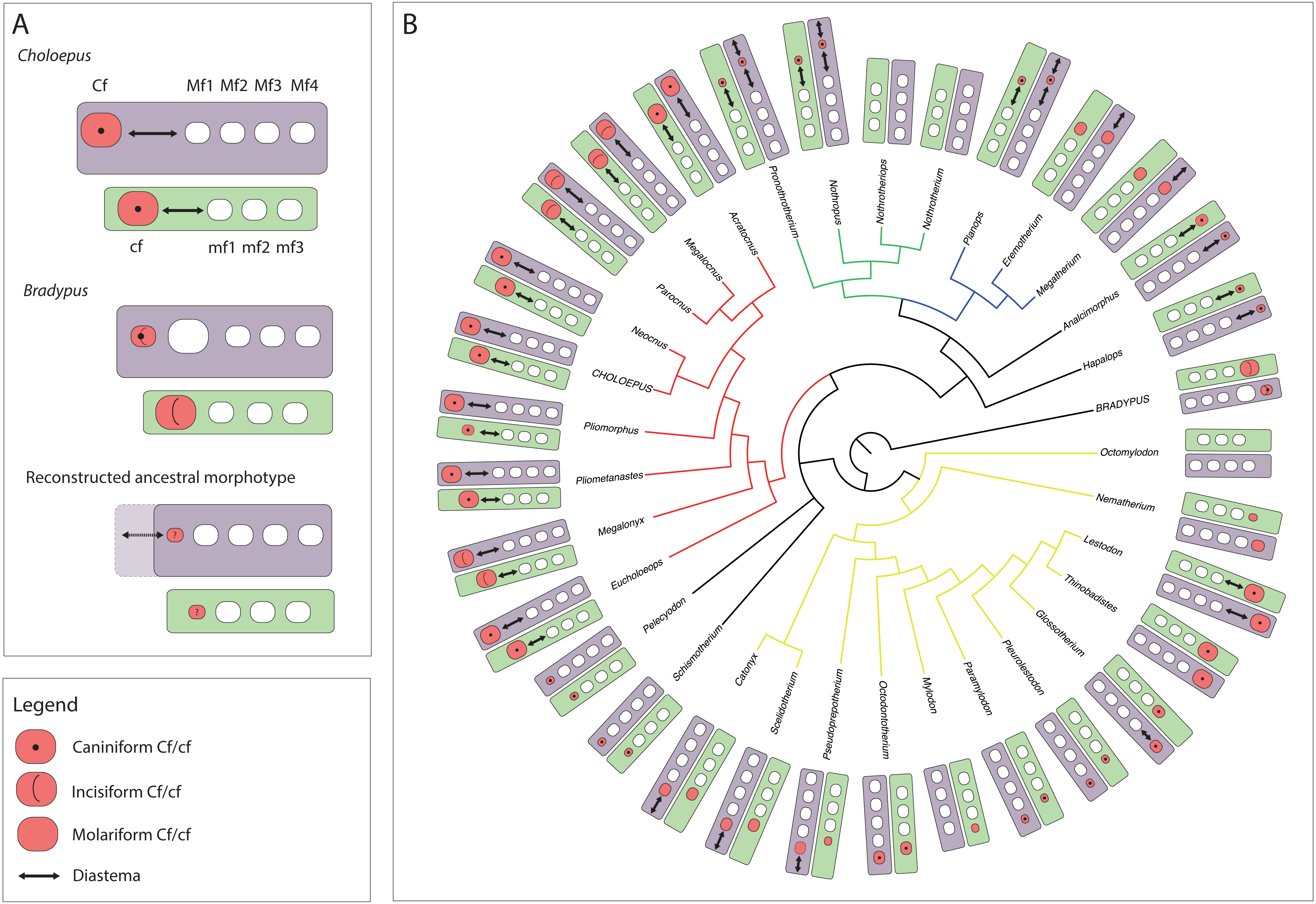

A
Choloepus
Cf
Mf1 Mf2 Mf3 Mf4
cf
mf1 mf2 mf3
Bradypus
Reconstructed ancestral morphotype
?
?
Legend
Caniniform Cf/cf
Incisiform Cf/cf
Molariform Cf/cf
Diastema
B
Pronothrotherium
Nothropus
Nothrotheriops
Nothrotherium
Planops
Eremotherium
Megatherium
Analcimorphus
Hapalops
BRADYPUS
Octomylodon
Nematherium
Lestodon
Thinobadistes
Glossotherium
Pleurolestodon
Paramylodon
Mylodon
Octodontotherium
Pseudoprepotherium
Scelidotherium
Catonyx
Schismotherium
Pelecyodon
Eucholoeops
Megalonyx
Pliometanastes
Pliomorphus
CHOLOEPUS
Neocnus
Parocnus
Megalocnus
Acratocnus
